# CD200 is overexpressed in the pancreatic tumor microenvironment and predictive of overall survival

**DOI:** 10.1007/s00262-024-03678-6

**Published:** 2024-04-15

**Authors:** Jessica Wedig, Shrina Jasani, Debasmita Mukherjee, Hannah Lathrop, Priya Matreja, Timothy Pfau, Liliana D’Alesio, Abigail Guenther, Lexie Fenn, Morgan Kaiser, Molly A. Torok, Jake McGue, Gina M. Sizemore, Anne M. Noonan, Mary E. Dillhoff, Bradley W. Blaser, Timothy L. Frankel, Stacey Culp, Phil A. Hart, Zobeida Cruz-Monserrate, Thomas A. Mace

**Affiliations:** 1 The James Comprehensive Cancer Center, Ohio State University Wexner Medical Center, Columbus, USA; 2 Molecular, Cellular and Developmental Biology Program, The Ohio State University, Columbus, USA; 3 Department of Surgical Oncology, University of Michigan, Ann Arbor, USA; 4 Department of Radiation Oncology, The Ohio State University, Columbus, USA; 5 Department of Internal Medicine, Division of Medical Oncology, The Ohio State University Wexner Medical Center, Columbus, USA; 6 Department of Internal Medicine, Division of Surgical Oncology, The Ohio State University Wexner Medical Center, Columbus, USA; 7 Department of Internal Medicine, Division of Hematology, The Ohio State University Wexner Medical Center, Columbus, USA; 8 Department of Biomedical Informatics, The Ohio State University, Columbus, USA; 9 Department of Internal Medicine, Division of Gastroenterology, Hepatology, and Nutrition, The Ohio State University Wexner Medical Center, 420 W. 12th Ave., Columbus, OH 43210 USA

**Keywords:** Pancreatic cancer, CD200, Tumor microenvironment, Overall Survival

## Abstract

Pancreatic cancer is an aggressive disease with a 5 year survival rate of 13%. This poor survival is attributed, in part, to limited and ineffective treatments for patients with metastatic disease, highlighting a need to identify molecular drivers of pancreatic cancer to target for more effective treatment. CD200 is a glycoprotein that interacts with the receptor CD200R and elicits an immunosuppressive response. Overexpression of CD200 has been associated with differential outcomes, depending on the tumor type. In the context of pancreatic cancer, we have previously reported that CD200 is expressed in the pancreatic tumor microenvironment (TME), and that targeting CD200 in murine tumor models reduces tumor burden. We hypothesized that CD200 is overexpressed on tumor and stromal populations in the pancreatic TME and that circulating levels of soluble CD200 (sCD200) have prognostic value for overall survival. We discovered that CD200 was overexpressed on immune, stromal, and tumor populations in the pancreatic TME. Particularly, single-cell RNA-sequencing indicated that CD200 was upregulated on inflammatory cancer-associated fibroblasts. Cytometry by time of flight analysis of PBMCs indicated that CD200 was overexpressed on innate immune populations, including monocytes, dendritic cells, and monocytic myeloid-derived suppressor cells. High sCD200 levels in plasma correlated with significantly worse overall and progression-free survival. Additionally, sCD200 correlated with the ratio of circulating matrix metalloproteinase (MMP) 3: tissue inhibitor of metalloproteinase (TIMP) 3 and MMP11/TIMP3. This study highlights the importance of CD200 expression in pancreatic cancer and provides the rationale for designing novel therapeutic strategies that target this protein.

**Supplementary Information:**

The online version contains supplementary material available at 10.1007/s00262-024-03678-6.

## Introduction

Pancreatic cancer is the 3rd leading cause of cancer deaths in the USA and has a 5 year survival rate of 13% [1]. Pancreatic ductal adenocarcinoma (PDAC) is the most common type of pancreatic cancers, accounting for over 90% of cases [2–4]. The combination of systemic chemotherapy and surgery can be potentially curative; however, most patients (> 80%) have locally advanced or metastatic disease at cancer diagnosis, so this is not feasible [5, 6]. While there have been recent advances in options for systemic chemotherapy, responses are not universal and dose-limiting toxicities are common [7–10]. Moreover, the tumors can become resistant to chemotherapy within weeks after starting treatment, limiting the beneficial effects [11]. As a result, efforts have been focused on identifying genetic and molecular drivers of PDAC progression to target to develop efficacious therapeutics.

The PDAC tumor microenvironment (TME) is characterized by a desmoplastic reaction and heterogeneous stromal and immune populations [12]. One of the major stromal components of the PDAC TME is the presence of cancer-associated fibroblasts (CAFs), which are differentiated from pancreatic stellate cells (PSCs). Two major populations of CAFs have been identified in PDAC: inflammatory and myo-cancer-associated fibroblasts (iCAFs and myCAFs) [13, 14]. The iCAFs are characterized as expressing low levels of αSMA and high levels of the inflammatory cytokines IL-6 and IL-11 that can expand and promote the immunosuppressive function of myeloid populations, such as tumor-associated macrophages (TAMs) and myeloid-derived suppressor cells (MDSCs) [14–16]. These myeloid cells are the most prominent immune populations in the pancreatic TME and can inhibit T cell effector function and proliferation and promote Treg differentiation through the secretion of immunosuppressive factors, such as IDO1 and Arg1 [17, 18]. The myCAFs are characterized as expressing high levels of αSMA and are involved in promoting a stiff extracellular matrix (ECM) comprised of collagen and fibronectin that can contribute to resistance to chemotherapy and limit T cell proliferation and movement [14, 19–21]. Identification and characterization of upregulated genes and proteins on these heterogeneous populations may provide insights for targeting the TME to limit PDAC progression.

CD200 is a glycoprotein that is expressed in the stromal, epithelial, and tumor cells, though CD200 expression has also been reported on small subsets of T cells, B cells, and dendritic cells [22–25]. CD200 expression has been reported to be induced by NF-κB and ERK signaling pathways [26, 27]. It binds to its receptor, CD200R, which is expressed by Tregs and myeloid cells [22, 28]. Overexpression of CD200 has been associated with differential outcomes across cancer types. In some tumors, such as central nervous system tumors and breast cancer, CD200 overexpression has been associated with a pro-tumorigenic effect [29, 30]. However, there have been studies in a melanoma model that have shown that CD200 expression in melanoma has an anti-tumor effect [31, 32]. Our laboratory previously reported that CD200 is overexpressed in patients with PDAC and that blockade of CD200 in a murine subcutaneous and genetically engineered mouse models reduced tumor burden, suggesting a pro-tumor role for CD200 in the context of pancreatic cancer [33]. Previously, we showed that CD200 is expressed by PDAC cell lines and in the stromal compartments of PDAC tissue specimens [33]. However, exactly which stromal and tumor populations in the pancreatic TME express CD200 has yet to be determined.

In addition to existing as a cell-surface protein, the ectodomain of CD200 can be cleaved by matrix metalloproteinases (MMPs) and a disintegrin and metalloproteinase (ADAM) proteins [34, 35]. The resulting soluble CD200 (sCD200) can be readily measured in plasma samples [35, 36]. Importantly, the CD200 ectodomain is still functional and thus can still interact with its receptor and initiate an immunosuppressive response [37]. Furthermore, in chronic lymphocytic leukemia (CLL), patients with higher sCD200 levels have worse overall survival (OS) and progression-free survival (PFS) than patients with lower sCD200 [38]. In PDAC, it is unknown if sCD200 is detectable in the plasma, which MMPs are involved, and if circulating levels of sCD200 correlate with worse OS and PFS in patients.

We hypothesize that CD200 protein and mRNA transcripts are upregulated by the tumor and stroma cells in the PDAC TME. Furthermore, we hypothesize that sCD200 is upregulated in PDAC and correlates to OS and PFS. Here, we investigate where CD200 is spatially expressed in the pancreatic TME and identify CD200 + cell populations. Finally, we investigate the mechanism of CD200 cleavage in PDAC and the correlation between sCD200 and OS and PFS in metastatic PDAC.

## Methods

### Multiplex immunofluorescence of PDAC specimens

Human tissue microarrays (TissueArray.com, PA961f and PA807) containing pancreatic tumors (*n* = 127), pancreatic tumor-adjacent tissue (*n* = 27), and normal pancreatic tissue (*n* = 18) were stained according to the protocol described previously [39, 40]. In brief, tissues were prepped on a slide warmer at 60 °C for 1 h, followed by three 10 min washes in xylene to remove paraffin. Tissue was rehydrated by 10 min washes in 100%, 90%, and 75% ethanol, followed by a 2 min wash with deionized water. Slides were fixed with 10% neutral buffered formalin for 30 min. Slides were blocked in BLOXALL (Vector, SP-6000) for 10 min. The primary antibodies for αSMA (Abcam, ab5694), CD45 (Abcam, ab10558), and FAP (Abcam, ab53066) were diluted 1:100. Primary antibody for PDGFRβ (Abcam, ab32570) was diluted 1:300, primary antibody for CD200 (Abcam, ab203887) was diluted 1:75, and primary antibody for PanCK (DAKO, M3515) was diluted 1:50. For each round of staining, slides were incubated with primary antibody for 1 h. DAPI (AKOYA Biosciences, FP1490) was diluted by adding 3 drops into 1 mL TBST. Slides were stained with DAPI for 10 min. 3 drops of Mouse/Rabbit (AKOYA Biosciences, ARH1001EA) secondary antibody were used for 10 min. Opals (AKOYA Biosciences, NEL861001KT) 480, 520, 570, 620, 690 were diluted 1:50; TSA-DIG was diluted 1:100; and 780 was diluted 1:25. Slides were stained with opals 480, 520, 570, 620, 690, and TSA-DIG for 10 min. Slides were stained with opal 780 for 1 h. Antigen-Retrieval was accomplished using AR6 (AKOYA, AR600250ML) and AR9 (AKOYA, AR900250ML) buffers followed by microwaving for 45 s at 100% power and 15 min at 20% power. After completing all rounds of staining, slides were mounted using ProLong™ Diamond Antifade Mountant (Invitrogen, P36970). Slides were imaged using Vectra Polaris and analyzed with QuPath.

### Pancreatic stellate cell isolation and nanostring analysis

Human PDAC tissues were obtained from ten patients (PSC1-PSC10) undergoing surgical resection at The Ohio State University Wexner Medical Center. The tissue was dissected into 0.5–1 mm^3^ pieces and plated in six-well 10 cm^2^ uncoated culture wells in DMEM (10% FBS, 1% AA) for two to three weeks. PSCs grew out of the pancreas and were characterized by morphology and αSMA expression. PSCs were maintained in culture for three passages. Human fetal primary pancreatic fibroblast cell lines were used as a control for normal PSCs and were obtained from Vitro Biopharma and cultured in MSC-GRO media with antibiotics. RNA was collected from patient PSCs (PSC1-PSC10) and control fibroblast cell lines via TRIzol extraction. RNA was analyzed using the nCounter PanCancer Immune Profiling Panel (Nanostring Technologies, Seattle Washington).

### Western blot

Harvested PSC1, PSC2, PSC3, PSC4, and PSC5 cells were incubated in lysis buffer (RIPA buffer, 1:100 protease inhibitor, 1:100 phosphatase inhibitor) for 1 h, after which sample buffer/mercaptoethanol (1:100 mercaptoethanol in Laemmli buffer) was added. Samples were then boiled for 10 min. Samples were run on an SDS–polyacrylamide gel (BioRad Mini Protean Tgx gradient gels) followed by transfer to nitrocellulose membranes (BioRad). Proteins were blocked in Tris-buffered saline containing 0.5% BSA. Primary antibodies used: rabbit anti-human CD200 (Abcam, ab203887) and mouse anti-human β-actin loading control (Invitrogen, MA5-15739). IRDye-fluorescent secondary antibodies for goat anti-rabbit (LiCOR, 926-32211) and goat anti-mouse (LiCOR, 926-68070) were used. Images for protein bands were obtained using LiCOR Odyssey CLx and analyzed with ImageStudio.

### Single-cell RNA-sequencing dataset analysis

Publicly available single-cell RNA-sequencing data (accession number GSE129455) on PDAC patient tumor samples from NIH dbGaP [41] were used to evaluate CD200 expression on myCAFs and iCAFs, and MMP3 and MMP11 expression in the pancreatic TME. Volcano plots were generated using Molecular and Genomics Informatics Core (MaGIC) Volcano Plot Tool, and pathway analysis was done via the Reactome pathway database.

### Cytometry by time of flight (CyTOF)

Peripheral Blood Mononuclear Cells (PBMCs) were obtained from additional research participants with PDAC and healthy donors using a density gradient centrifugation method with Ficoll-Paque (Pharmacia Biotech, 171440-03). Isolated PBMCs were lysed with Red Blood Cells lysis buffer and then washed with Maxpar Cell Staining Buffer (Standard Biotools, 201068). 3 × 10^6^ cells were aliquoted per sample and treated with 5 uL of Human TruStain FcX (BioLegend, 422302) for 10 min. Samples were then surfaced stained with metal-isotope bound antibodies (Supplemental Table 1) from the Maxpar Direct Immune Profiling Assay kit (Standard Biotools, 201334) with the addition of antibodies for CD11b (Standard Biotools, 3209003B), CD33 (BioLegend, 303419), and CD200 (BioLegend, 329219) followed by three washes in Maxpar Cell Staining Buffer. Cells were then stained with intercalation solution (Cell-ID Intercalator-Ir (Standard Biotools, S00093) diluted to 125 nM in Maxpar Fix and Perm (Standard Biotools, S00092)). After staining, samples were washed twice with Maxpar cell staining buffer, followed by two washes with EDTA diluted to 5 uM in deionized H_2_O. PBMCs were transferred to filter cap flow tubes. Maxpar acquisition solution (Standard Biotools, 201248) and EQ Four Element Calibration beads (Standard Biotools, 201078) were added to the samples prior to being run through the Helios Mass Cytometer. Data were uploaded to and analyzed with OMIQ.

### Enzyme-linked immunosorbent assay (ELISA)

Blood samples were obtained from patients with metastatic PDAC who previously participated in a randomized clinical trial (RCT) [NCT01280058]. In this phase 2, RCT participants were randomized to receive pelareorep (a proprietary oncolytic reovirus under investigation as a cancer therapeutic) + carboplatin/paclitaxel or carboplatin/paclitaxel alone [42]. There were no differences in clinical outcomes, including PFS or OS, between the two trial arms. Baseline plasma samples collected prior to receiving therapy were selected for the current analysis. sCD200 in the plasma and pancreatic cell supernatants was measured using a human CD200 ELISA kit (Invitrogen by Thermo Fisher Scientific, EHCD200) which was run according to the manufacturer’s instructions. MMP3, MMP11, and TIMP3 in PDAC participants were measured by human MMP3 (R&D Systems, DMP300), Human MMP11 (LSBio, LS-F21105-1), and human TIMP3 (Thermo Fisher, EH458RB) ELISA kits which were run according to the manufacturer’s instructions. The median sCD200 level was calculated in participants that had detectable sCD200 (defined 40 pg/mL or greater; this value represents the lower detection limit of the kit). Concentrations above the median were defined as “high,” and values below the mean were classified as “low.” Quantified MMP3, MMP11, and TIMP3 were used to calculate patient ratios of circulating MMP3/TIMP3 and MMP11/TIMP3, and the ratios were correlated to sCD200 plasma concentrations.

### Statistics

Analysis of variance using Tukey’s test for pairwise comparisons was used to compare CD200 by pathology diagnosis (normal, cancer adjacent, pancreatic cancer), pathology grade (1, 2, 3), and cancer stage (I, II, and III/IV), while independent-samples t tests were used to compare CD200 in relation to median age (i.e., < 53 yrs vs. ≥ 53 yrs) and sex. Independent-samples *t* tests were also used to compare CD200 MMI between PDAC subjects and healthy controls for myeloid populations and specific T cell populations as well as to compare CD200 expression between PSCs derived from PDAC patients and healthy controls. Kaplan–Meier curves were used to examine the differences in overall survival and progression-free survival between those with detectable sCD200 (> 40 pg/mL) and those with undetectable sCD200 and then between those with sCD200 in the upper 25th percentile and those in the lower 75th percentile. Log-rank tests were used to compare survival between the groups. Scatterplots were constructed to examine the relationship between sCD200 and each of the following: MMP3, MMP11, MMP3/TIMP3, and MMP11/TIMP3, and Spearman’s ρ correlation coefficient was calculated to quantify the strength and direction of the associations. Figures were made using GraphPad Prism 10.

## Results

### CD200 is overexpressed in the pancreatic TME

**Fig. 1:**
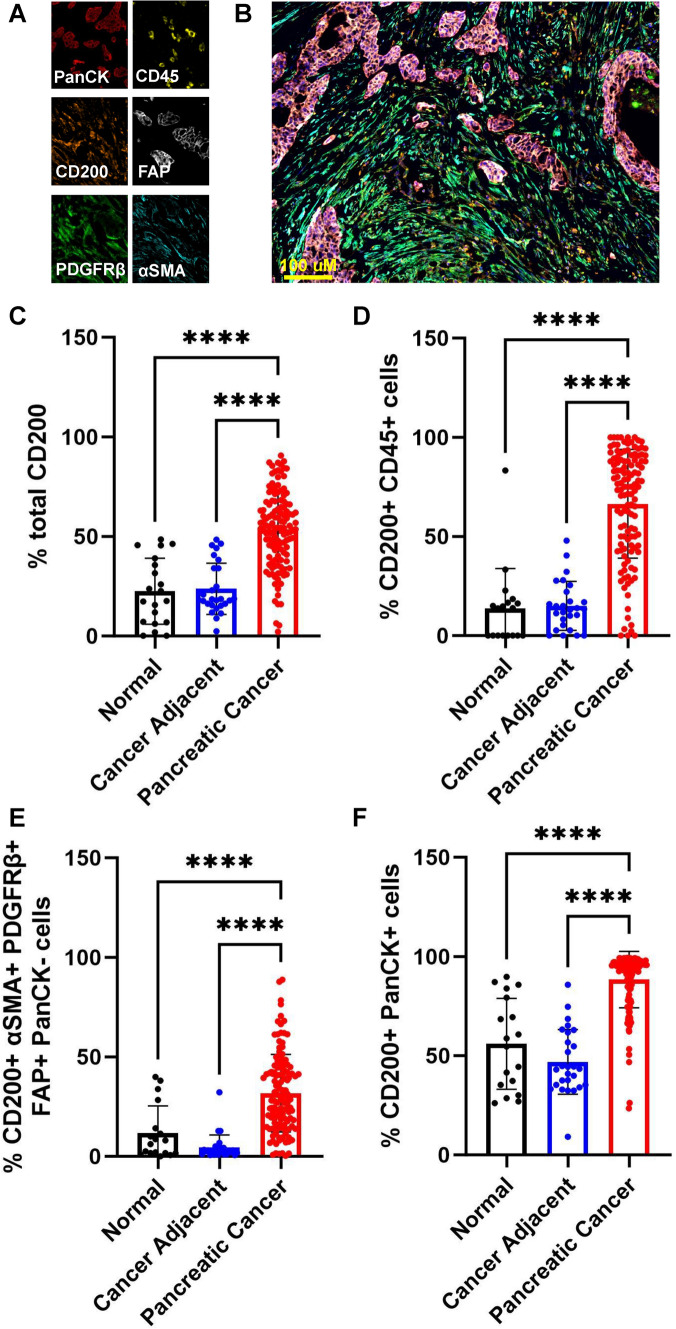
CD200 is upregulated in the pancreatic TME. Tissue microarrays were stained for **A** tumor (PanCK), immune (CD45), stroma (FAP, PDGFRβ, αSMA), and CD200. **B** Representative image of full multiplex IF panel. CD200 expression in normal (*n* = 18), cancer-adjacent (*n* = 27), and pancreatic cancer (*n* = 127) tissues were quantified in QuPath as **C** % total CD200 **D** % CD200 + CD45 + immune cells, **E** % CD200 + αSMA + PDGFRβ + FAP + PanCK- stromal cells, and **F** % CD200 + PanCK + epithelial cells. Means ± STD, **** = *p* < 0.0001

We previously reported that CD200 is elevated in PDAC [33], and we hypothesized that CD200 was overexpressed on both tumor and stromal cells in the pancreatic TME. To further investigate this, we stained pancreatic cancer tissue (*n* = 127) specimens from different stages of disease progression, sex, and age (Supplemental Table 2) along with cancer-adjacent (*n* = 27) and normal tissues (*n* = 18) with a panel for multiplex IF focusing on stromal markers (Fig. 1A & B). Stromal populations were identified as expressing one or a combination of αSMA, PDGFRβ, and FAP and were negative for pan cytokeratin (PanCK). CD45 + only cells were identified as immune cells and PanCK expression was used to identify epithelial cells and tumor cells of epithelial origin. Malignant tissues significantly expressed more CD200 overall than the cancer-adjacent and normal pancreatic tissues (Fig. 1C). When looking at CD200 across different cell populations in the pancreatic TME, we observed that CD200 expression was increased on immune populations (Fig. 1D), stromal populations (Fig. 1E), and epithelial cells (Fig. 1F). We also found that total CD200 expression was not affected by pathology grade (Supplemental Fig. 1A), nor did pathology grade impact CD200 expression on immune (Supplemental Fig. 1B) or stromal (Supplemental Fig. 1C) cells. CD200 expression was significantly decreased on tumor cells in grade 3 tumors (Supplemental Fig. 1D). Similarly, when examining if CD200 expression in the TME was affected by disease stage, we observed an insignificant difference in total CD200 expression (Supplemental Fig. 1E) as well no significant difference in CD200 expression on immune (Supplemental Fig. 1F), stromal (Supplemental Fig. 1G), and tumor cells (Supplemental Fig. 1H). When looking at age, we observed no significant difference in CD200 expression in younger patients compared to older patients (Supplemental Fig. 1I-L). We also compared CD200 expression in the pancreatic TME of females and males. There was an insignificant difference in CD200 expression between female and male patients (Supplemental Fig. 1M-P). Ultimately, these results indicate that CD200 is overexpressed by tumor, stromal cells, and immune populations in the PDAC TME, and that CD200 expression is not dependent on pathology grade or cancer stage.

### CD200 is upregulated by iCAFs in the pancreatic TME

**Fig. 2:**
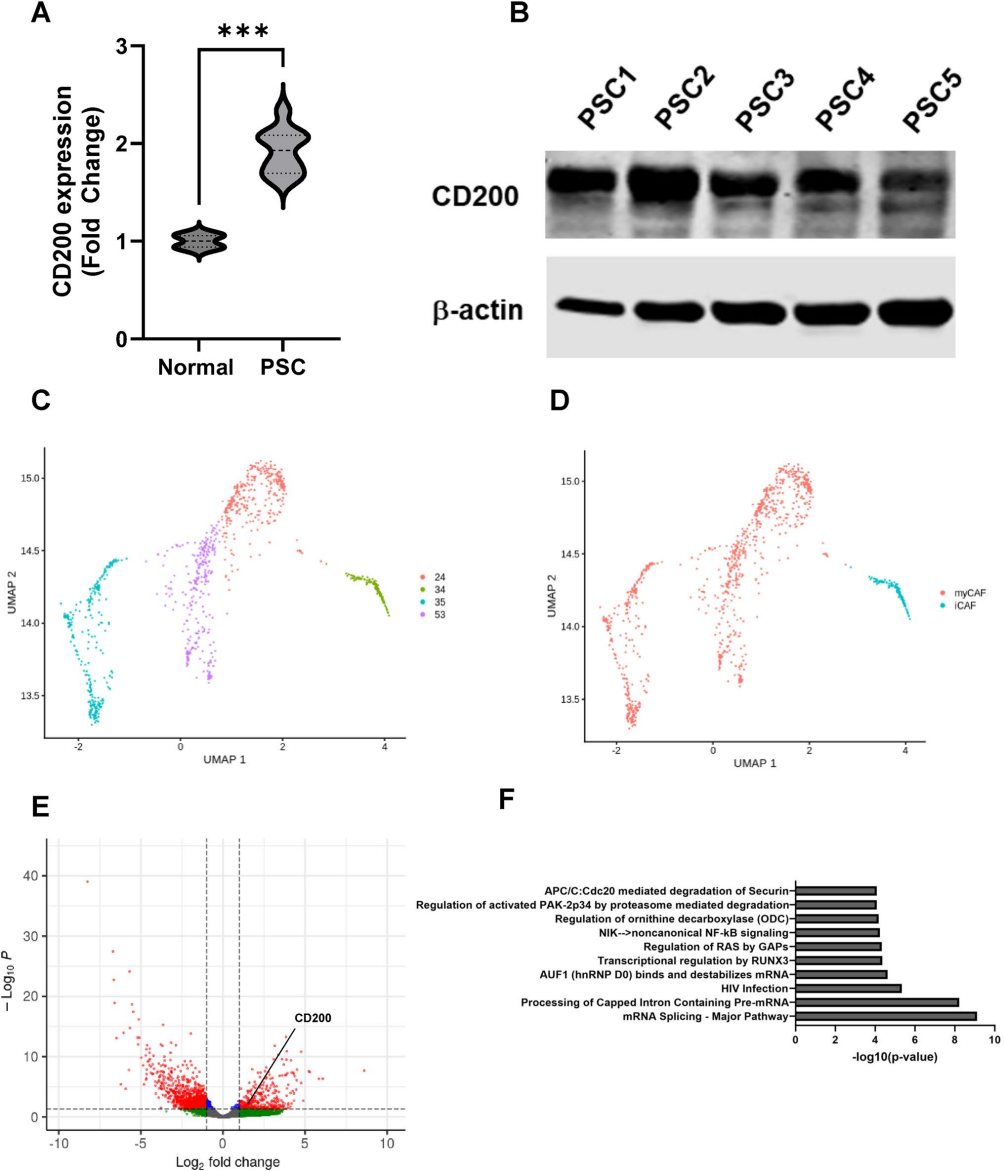
CD200 expression is upregulated on iCAFs. **A** CD200 mRNA expression from PDAC patient PSCs compared to normal HPPFC controls via Nanostring analysis. **B** Western Plot showing CD200 expression in PDAC patient PSCs. **C** UMAP of CAF clusters from single-cell RNA-sequencing dataset of PDAC patients from NIH dbGaP and **D** identification of clusters as myCAFs and iCAFs. **E** Volcano-plot showing significantly upregulated and downregulated genes in iCAFs. **F** Top 10 pathways significantly affected in iCAFs via Reactome analysis. *** = *p* < 0.001

Our multiplex IF identified that CD200 was expressed by stromal cells in the PDAC TME. We confirmed that PDAC-derived PSCs had upregulated CD200 mRNA (Fig. 2A) and protein (Fig. 2B) expression compared to normal pancreas fibroblast controls. However, the stromal cells of the PDAC TME are heterogeneous, comprising of PSCs, iCAFs, and myCAFs. Thus, we investigated whether CD200 expression was limited to specific subsets of stromal populations. We analyzed publicly available single-cell RNA-seq data [41] of PDAC tissue collected from patients. We identified 4 clusters of CAFs (Fig. 2C). Genetic markers to distinguish iCAFs and myCAFs (Supplemental Table 3) were used to further identify clusters 24, 35, and 53 as myCAFs and cluster 34 as iCAFs (Fig. 2D). Analysis of genes on the iCAFs revealed that CD200 was significantly upregulated on iCAFs (Fig. 2E) and that iCAFs had significant changes in the NFκB and RUNX3 pathways as well as pathways related to mRNA processing (Fig. 2F). These data indicate that CD200 is more highly expressed on iCAFs in PDAC.

### CD200 expression on immune populations

**Fig. 3:**
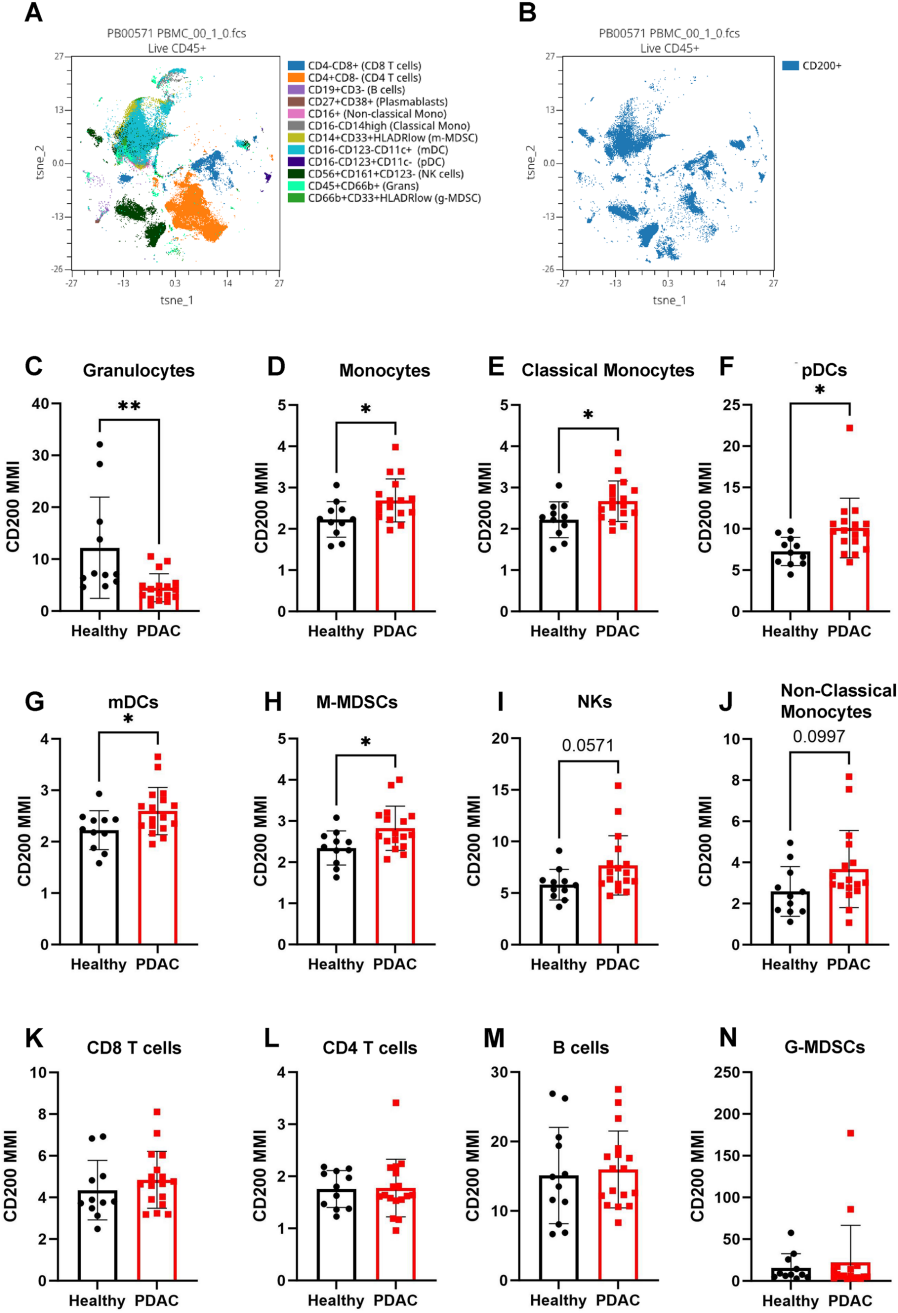
CD200 is upregulated on myeloid populations in the blood of patients with PDAC. PBMCs were isolated from PDAC patients (*n* = 17) and healthy donors (*n* = 12) and stained with a panel of 38 antibodies for CyTOF. **A** A tSNE showing immune populations and **B** CD200 expression across immune populations. Mean metal intensity (MMI) of CD200 was quantified on **C** granulocytes, **D** monocytes, **E** classical monocytes, **F** pDCs, **G** mDCs, **H** M-MDSCs, **I** NK, **J** Non-classical monocytes, **K** CD8 T cells, **L** CD4 T cells, **M** B cells, and **N** G-MDSCs. Means ± STD, * = *p* < 0.05

While we have observed CD200 expression on the tumor and stromal cells in the PDAC TME, CD200 has been reported to be expressed on immune populations, such as T cells [23], B cells [24], and DCs [25]. To investigate changes in CD200 expression on immune populations in PDAC, we designed a CyTOF panel to analyze over 37 different immune populations (Supplemental Fig. 2) on PBMCs of PDAC patients (*n* = 17) undergoing surgical resection and healthy/control individuals (*n* = 12) as previously described [43]. We identified circulating immune populations (Fig. 3A) and quantified their CD200 expression (Fig. 3B). We observed that PDAC patients had significantly decreased CD200 expression on granulocytes (Fig. 3C). CD200 expression was significantly increased on monocytes (Fig. 3D), classical monocytes (Fig. 3E), plasmacytoid DCs (pDCs) (Fig. 3F), myeloid DCs (mDCs) (Fig. 3G), and monocytic MDSCs (M-MDSCs) (Fig. 3H). We also observed a general increase in NK cells (Fig. 3I) and non-classical monocytes (Fig. 3J). However, we observed no significant difference in CD200 expression on CD8 T cells (Fig. 3K), CD4 T cells (Fig. 3L), B cells (Fig. 3M), and granulocytic MDSCs (G-MDSCs) (Fig. 3N). Our CyTOF panel also allowed for us to compare CD200 expression on specific T cell populations in PDAC patients compared to healthy individuals. PDAC patients did not have a significant change in CD200 expression on naïve (Supplemental Fig. 3A), activated (Supplemental Fig. 3B), CM (Supplemental Fig. 3C), EM (Supplemental Fig. 3D), or TE (Supplemental Fig. 3E) CD8 T cells. However, on CD4 T cells, we observed a significant increase in EM CD4 T cells (Supplemental Fig. 3F), and no significant change in naïve (Supplemental Fig. 3G), activated (Supplemental Fig. 3H), CM (Supplemental Fig. 3I), or TE (Supplemental Fig. 3J) CD4 T cells. We also observed no significant difference in CD200 expression on Tregs (Supplemental Fig. 3K), Th1 (Supplemental Fig. 3L), Th2 (Supplemental Fig. 3M), or Th17 cells (Supplemental Fig. 3N). This indicates that CD200 expression is upregulated on myeloid-derived immune populations in PDAC, and that there is no significant change in CD200 on CD8 T cell populations in PDAC.

### PDAC patients with high sCD200 have worse survival

**Fig. 4:**
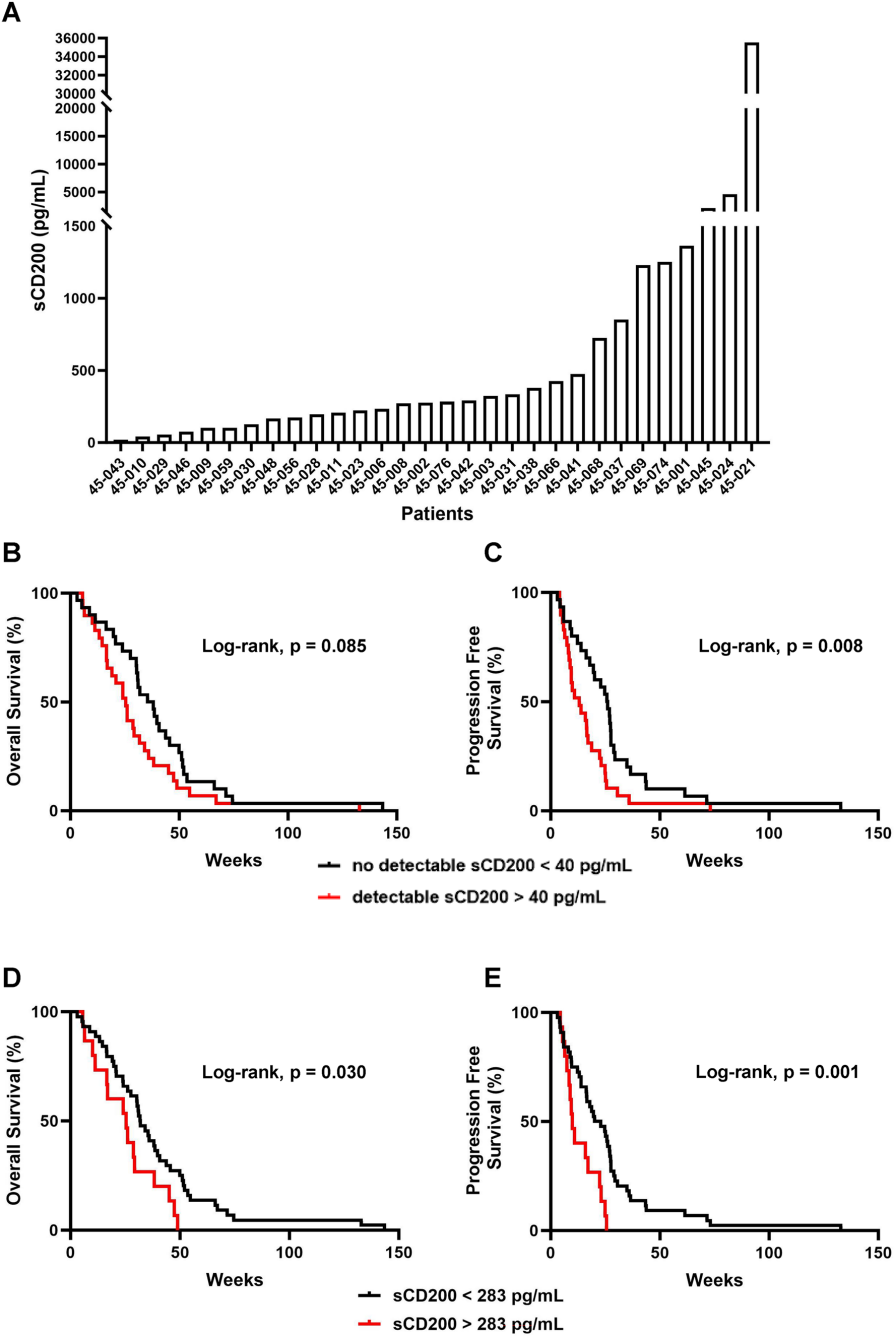
Higher sCD200 is associated with worse survival in PDAC patients. **A** Waterfall plot of sCD200 expression in plasma from patients with metastatic PDAC measures with ELISA. Survival curves for **B** OS and **C** PFS in patients with detectable sCD200 (*n* = 29) compared to patients with undetectable sCD200 (*n* = 30). Survival curves for **D** OS and **E** PFS in patients with high sCD200 (*n* = 15) compared to patients with low sCD200 (*n* = 44)

CD200 can be cleaved and expressed as sCD200 in the plasma in patients with CLL [38]; however, this has not been explored in PDAC. Thus, we investigated whether sCD200 levels were elevated in metastatic PDAC. We confirmed via ELISA that PDAC patients can express sCD200 in their plasma, though there is intersubject variability in concentrations (Fig. 4A). Due to the variance of sCD200 levels, we hypothesized that the amount of sCD200 correlated with OS and PFS in PDAC. We compared OS and PFS of patients that had detectable sCD200 (*n* = 29) to patients that did not have detectable sCD200 (*n* = 30) and observed worse OS when sCD200 was detectable (Fig. 4B) and significantly worse PFS (Fig. 4C). We further decided to look at how high sCD200 correlates with survival. We observed that patients that have high sCD200 (*n* = 15) levels have significantly worse OS (Fig. 4D) and PFS (Fig. 4E) than patients with low or non-detectable sCD200 (*n* = 44). Ultimately, these data suggest that sCD200 can be predicative of worse survival outcomes in patients with metastatic PDAC.

### MMP3 and MMP11 levels in patients with PDAC patients correlate with sCD200 levels

**Fig. 5:**
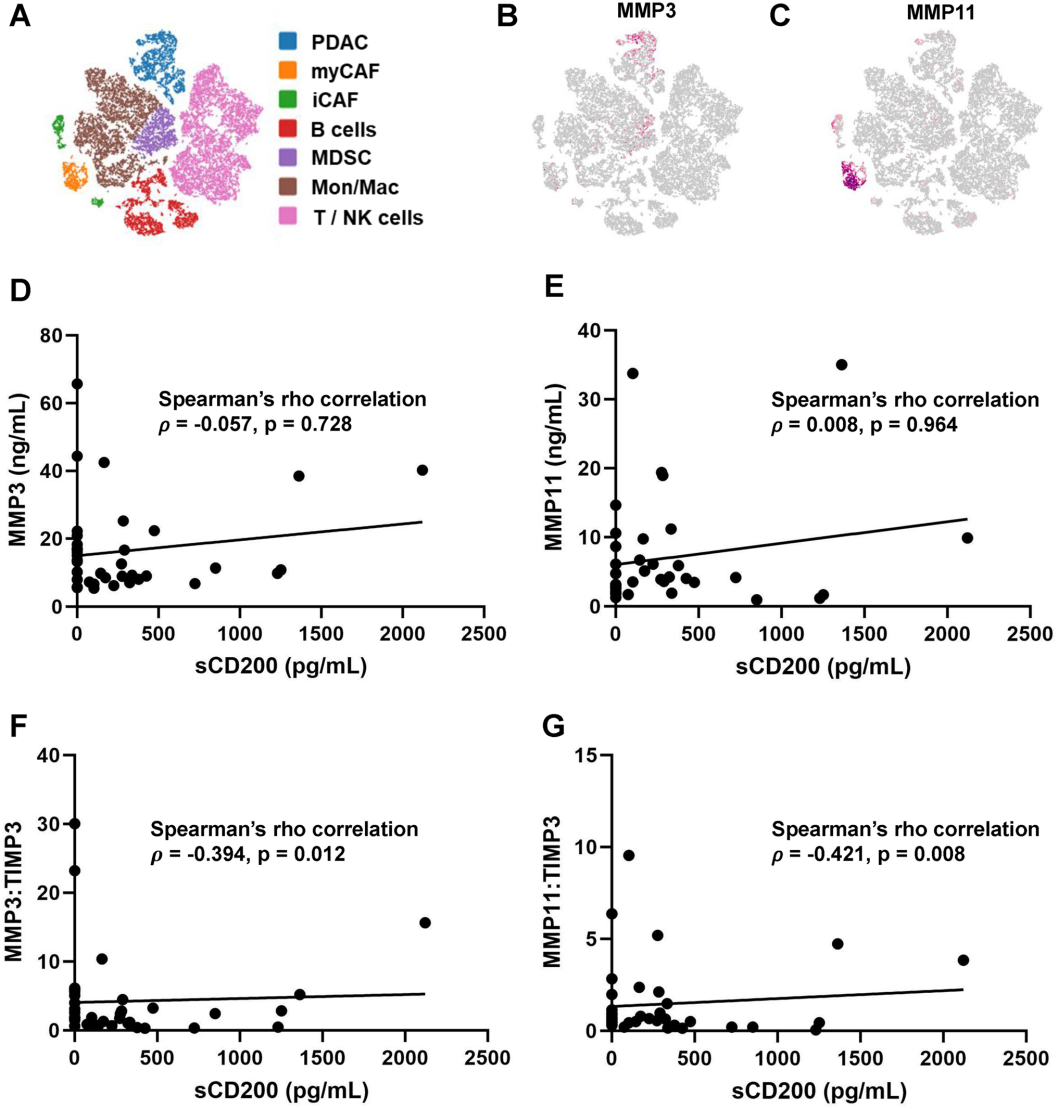
MMP3 and MMP11 correlate with sCD200. **A** tSNE of populations in pancreatic TME identified by single-cell RNA-sequencing dataset of PDAC patients from NIH dbGaP and expression of **B** MMP3 and **C** MMP11 in the PDAC TME. ELISA on plasma from metastatic PDAC patients (*n* = 40) targeting MMP3, MMP11, and TIMP3 expression. **D** MMP3 expression and **E** MMP11 expression correlated with sCD200 levels. **F** MMP3/TIMP3 and **G** MMP11/TIMP3 ratios correlated with sCD200 levels

MMP3 and MMP11 have previously been shown to be involved in CD200 ectodomain shedding in basal cell carcinoma [34] and thus may be involved in regulating the cleavage of CD200 in PDAC. Single-cell RNA-sequencing of cell populations from the NIH dbGaP [41] in the PDAC TME (Fig. 5A) indicates that MMP3 is expressed in the tumor (Fig. 5B) and MMP11 is expressed in the stromal populations (Fig. 5C). Since both MMP3 and MMP11 are expressed in the pancreatic TME and can cleave the ectodomain of CD200, we hypothesized that the circulating levels of MMP3 and MMP11 in patients with PDAC correlated with the circulating levels of sCD200. To investigate this, we performed ELISAs to quantify the concentration of MMP3 and MMP11 in the plasma of patients with PDAC, which we then correlated to the sCD200 levels. There was no significant correlation between the circulating levels of MMP3 (Fig. 5D) or MMP11 (Fig. 5E) and sCD200 in PDAC. We then hypothesized that factors that regulate MMP3 and MMP11 enzymatic activity, such as expression of TIMP3 [35, 44], may be contributing to the lack of correlation observed. Since TIMP3 can inhibit MMP3 and MMP11 cleavage activity, we performed an ELISA to quantify TIMP3, then quantified the ratio of MMP3/TIMP3 and MMP11/TIMP3, and ultimately correlated these ratios to sCD200 levels. We observed that patients with a higher MMP3/TIMP3 (Fig. 5F) ratio or MMP11/TIMP3 (Fig. 5G) ratio had a significant correlation with sCD200 levels in PDAC. These results suggest that sCD200 correlates with MMP3 and MMP11 levels.

## Discussion

We previously reported CD200 is expressed in the pancreatic TME [33], and in this report, we are the first to show that CD200 is overexpressed on stromal, immune, and epithelial cells compared to cancer-adjacent tissues and normal tissues. However, an important limitation to our studies is that we were not able to analyze tissue that contain invasive edges. Future staining of invasive edges for CD200 expression may reveal whether or not CD200 plays a role for pancreatic cancer invasion and progression. Furthermore, staining of the tissue only indicates which cell types express CD200 in the pancreatic TME. Future analyses using newer techniques such as spatial transcriptomics could provide valuable information to the function of these CD200 + cell types and their interactions in the tumor.

Importantly, this is the first demonstration that CD200 is expressed on iCAFs in PDAC. iCAFs have a secretory phenotype and can secrete inflammatory cytokines and chemokines [14, 15]. In particular, iCAFs secrete SDF-1 and MCP-1 [15]. SDF-1 and MCP-1 have previously been shown to be chemoattractants for MDSCs and other myeloid cells through the SDF-1/CXCR4 axis [45] and MCP-1/CCR2 axis [46], respectively. The CD200 + iCAFs would be able to interact directly with the recruited myeloid cells, which express the receptor CD200R [33]. As CD200-CD200R signaling has been previously reported to promote an immunosuppressive phenotype in dendritic cells and macrophages [47, 48], the interaction between CD200 + iCAFs and CD200R + myeloid cells may be a mechanism that promotes an immunosuppressive TME in pancreatic cancer. Thus, further studies analyzing whether CD200 + iCAFs interact with CD200R + myeloid cells and promote their immunosuppressive activity is warranted.

In addition to having upregulated CD200, we have reported that iCAFs in PDAC have significant changes in in the NFκB and RUNX3 pathways as well as pathways related to mRNA processing and splicing. Importantly, the NFκB pathway has previously been shown to be involved in upregulating CD200 expression [26]. We also observed a significant difference in the RUNX3 pathway in iCAFs. Increased RUNX3 expression was previously shown to be increased in CD200 + CTLs and may also be correlative to CD200 expression in iCAFs [23]. RUNX3 has also previously been shown to be expressed in pancreatic cancer and to play a role in promoting immunosuppression [49, 50]. Interestingly, we also observed a significant difference in mRNA splicing pathways. CD200 has previously been shown to be affected by alternative splicing, which can result in a truncated version of CD200 [51]. This truncated CD200 has been shown to be a competitive antagonist to full-length CD200 [52]. Further investigation into the ratio of full-length CD200 expression vs truncated CD200 expression on iCAFs and how this ratio impacts iCAF function is warranted.

MDSCs and monocytes were both observed to be circulating in peripheral blood of PDAC patients and are associated with worse overall survival in PDAC [53, 54]. We are the first to report that CD200 expression increases on circulating monocytes, particularly classical monocytes, pDCs and mDCs, and M-MDSCs in patients with PDAC compared to healthy individuals. We have previously reported that the receptor CD200R is also significantly upregulated on MDSCs and that CD200 stimulation promotes their expansion [33]. Interactions between the circulating CD200 + myeloid cells and CD200R + MDSCs could be promoting the differentiation and expansion of immunosuppressive myeloid populations that could infiltrate into the tumor and promote an immunosuppressive TME. CD200 + CD8 + T cells were previously reported to be crucial for effective PD-1/PDL-1 blockade in murine tumor models [23]. Here, we did not observe any differences in CD200 expression on CD8 + T cells; furthermore, we previously reported that dual blockade of CD200 and PD-1 further reduces tumor burden in a subcutaneous pancreatic tumor model [33]. The discrepancy may be due to the abundance of myeloid populations in PDAC [55] and that CD200 signaling in myeloid cells is associated with the secretion of factors that mediate an immunosuppressive response on T cells [17, 18, 47, 48]. Our observation that CD200 expression increases on myeloid populations coupled with previous observations of myeloid populations (macrophages and MDSCs) being abundant in PDAC [55] may suggest that CD200 is promoting tumor progression through a myeloid-dependent mechanism. Thus, future studies should be conducted to specifically investigate the role of CD200 signaling in myeloid cells in PDAC.

The ectodomain of CD200 has been reported to be shed, and the resulting sCD200 has been reported in the plasma of patients with various types of cancers, including CLL and breast cancer [38, 56]. This is the first report to show that sCD200 is expressed in the plasma of PDAC patients. MMPs are secreted in the pancreatic TME and are involved in ECM remodeling, angiogenesis, metastasis. MMP3 and MMP11 are stromelysins that are involved in degrading ECM components [57] and their overexpression in PDAC is associated with worse survival [58, 59]. Both MMP3 and MMP11 have been shown to be capable of cleaving CD200 [34]. Though we reported no correlation between MMP3 and MMP11 plasma levels with sCD200 plasma levels in PDAC patients, we are the first to show a correlation of MMP3/TIMP3 and MMP11/TIMP3 plasma levels with sCD200 levels. However, our findings do not show causation, and further investigation is necessary to determine if MMP3 and MMP11 directly contribute to the sCD200 levels observed in patients. In addition, this study is not comprehensive of all MMPs and ADAM proteins that may be involved in CD200 ectodomain shedding in PDAC. For example, in CLL, ADAM28 is involved in CD200 ectodomain shedding [35], and other MMPs and ADAM proteins are involved in regulating protein shedding [60, 61], and thus, more comprehensive analyses on how CD200 ectodomain shedding is regulated are warranted. In addition, as there was a wide range of sCD200 expression in patients with metastatic PDAC, it would be worthwhile to expand the population size to further understand sCD200 correlation with MMP3 and MMP11.

Finally, we are the first to report that sCD200 is expressed in the plasma in metastatic PDAC patients and, importantly, that high levels of circulating sCD200 are associated with worse OS and PFS. This study is limited as only the plasma from metastatic patients were used to evaluate sCD200. Future investigations into whether sCD200 levels are impacted by cancer stage and if it is also a prognostic biomarker in earlier stages in PDAC are warranted. sCD200 has been reported to be fully functional and thus is still capable of interacting with the receptor bound on the surface of myeloid cells [37]. The circulating sCD200 in PDAC patients may be another avenue in which CD200R + myeloid cells can be stimulated and skewed towards an immunosuppressive phenotype, though this will need to be investigated in future studies. Ultimately, these results suggest that CD200 may be a biomarker for survival prognosis and may provide support for future investigations on targeting CD200 in PDAC.

### Supplementary Information

Below is the link to the electronic supplementary material.

Supplementary file 1 (PDF 1584 kb)
