# Supplementary material for: CD200 is overexpressed in the pancreatic tumor microenvironment and predictive of overall survival
Source: Cancer Immunol Immunother. 2024 Apr 15;73(6):96. doi: 10.1007/s00262-024-03678-6 (PMC11018596; doi:10.1007/s00262-024-03678-6)
Supplement: Supplementary file 1 — (PDF 1584 kb) [file 262_2024_3678_MOESM1_ESM.pdf]

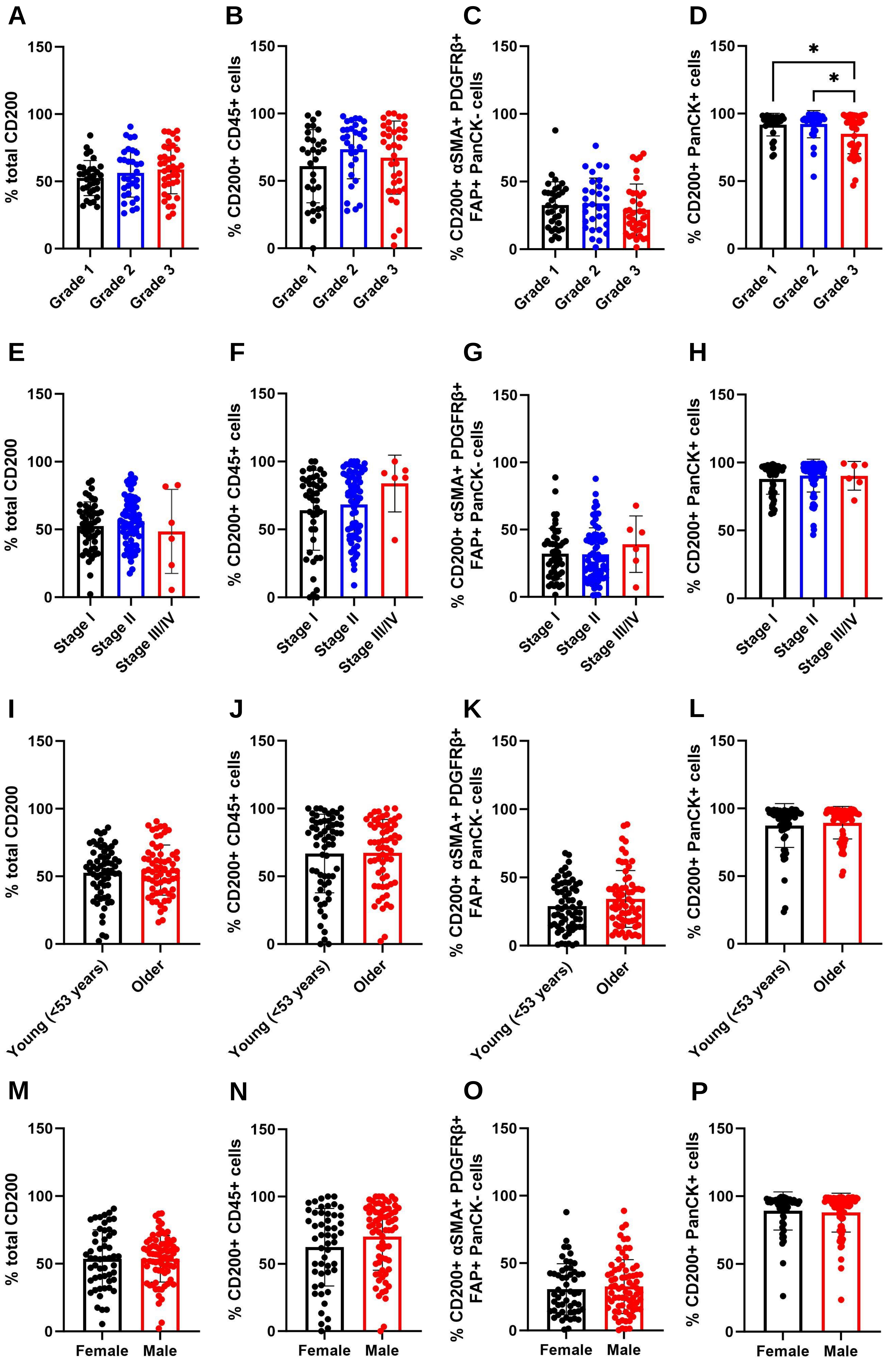
A
B
C
D
% total CD200
% CD200+ CD45+ cells
% CD200+ αSMA+ PDGFRβ+ FAP+ PanCK- cells
% CD200+ PanCK+ cells
150
100
50
0
Grade 1
Grade 2
Grade 3
*
*
E
F
G
H
Stage I
Stage II
Stage III/IV
I
J
K
L
Young (<53 years)
Older
M
N
O
P
Female
Male

Supplemental Figure 1: CD200 expression in the pancreatic tumor microenvironment is not dependent on pathology grade or cancer stage. Tissue microarrays were stained for tumor (PanCK), immune (CD45), stroma (FAP, PDGFRβ, αSMA), and CD200. CD200 expression was quantified as **A** % total CD200, **B** % CD200+ CD45+ immune cells, **C** % CD200+ αSMA+ PDGFRβ+ FAP+ PanCK- stromal cells, and **D** % CD200+ PanCK+ tumor cells in patients with grade 1 (n = 32), grade 2 (n = 31), and grade 3 (n = 37) tumors. CD200 expression was quantified as **E** % total CD200, **F** % CD200+ CD45+ immune cells, **G** % CD200+ αSMA+ PDGFRβ+ FAP+ PanCK- stromal cells, and **H** % CD200+ PanCK+ tumor cells in patients with stage I (n = 48), stage II (n = 71), and stage III/IV (n = 6) disease. CD200 expression was quantified as **I** % total CD200, **J** % CD200+ CD45+ immune cells, **K** % CD200+ αSMA+ PDGFRβ+ FAP+ PanCK- stromal cells, and **L** % CD200+ PanCK+ tumor cells in patients younger than the median age, 53 years, (n = 62) and patients older than the median (n = 65). CD200 expression was quantified as **M** % total CD200, **N** % CD200+ CD45+ immune cells, **O** % CD200+ αSMA+ PDGFRβ+ FAP+ PanCK- stromal cells, and **P** % CD200+ PanCK+ tumor cells in female (n = 53) and male (n = 74) patients. Means ± STD, * = $p < 0.05$

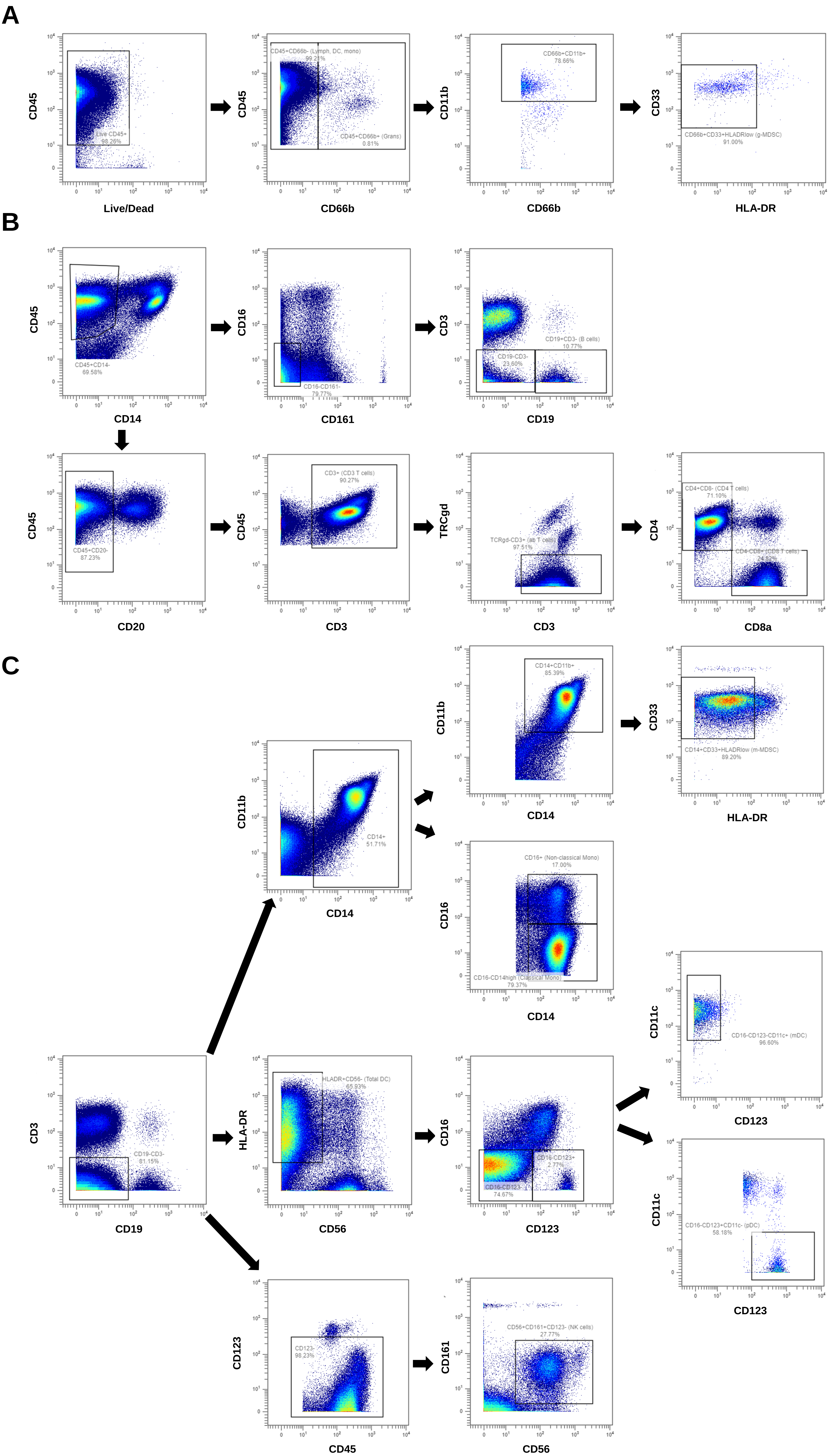
A
CD45
Live/Dead
Live CD45+ 98.26%
CD45+CD66b- (Lymph, DC, mono) 99.21%
CD45+CD66b+ (Grans) 0.81%
CD66b
CD11b
CD66b+CD11b+ 78.66%
CD33
HLA-DR
CD66b+CD33+HLADRlow (g-MDSC) 91.00%
B
CD14
CD45+CD14- 69.58%
CD16
CD161
CD16-CD161- 79.77%
CD3
CD19
CD19+CD3- (B cells) 10.77%
CD19-CD3- 23.60%
CD20
CD45+CD20- 87.23%
CD3+ (CD3 T cells) 90.27%
TRCgd
TCRgd-CD3+ (ab T cells) 97.51%
CD4
CD8a
CD4+CD8- (CD4 T cells) 71.10%
CD4-CD8+ (CD8 T cells) 24.82%
C
CD14+ 51.71%
CD14+CD11b+ 85.39%
CD14+CD33+HLADRlow (m-MDSC) 89.20%
CD16+ (Non-classical Mono) 17.00%
CD16-CD14high (Classical Mono) 79.37%
CD19-CD3- 81.15%
HLA-DR
CD56
HLADR+CD56- (Total DC) 65.93%
CD123
CD16-CD123+ 2.77%
CD16-CD123- 74.67%
CD11c
CD16-CD123-CD11c+ (mDC) 96.60%
CD16-CD123+CD11c- (pDC) 58.18%
CD45
CD123- 98.23%
CD161
CD56+CD161+CD123- (NK cells) 27.77%

Supplemental Figure 2: Representative gating strategy on patient PBMCs. A Representative gating strategy for CD66b+ CD11b+ granulocytes and CD66b+ CD33+ HLADR^{low} g-MDSCs. **B** Representative gating strategy for CD19+CD3- B cells, CD8+ T cells, and CD4+ T cells. CD45+ CD14- cells gated off the CD45+ CD66b- population. **C** Representative gating strategy for CD14+ monocytes (CD16- classical and CD16+ nonclassical), CD14+ CD33+ HLADR^{low} m-MDSCs, CD16- CD123+ CD11c- pDCs, CD16- CD123- CD11c+ mDCs, and CD56+ CD161+ CD123- NK cells. CD19- CD3- cells gated off the CD45+ CD66b- population.

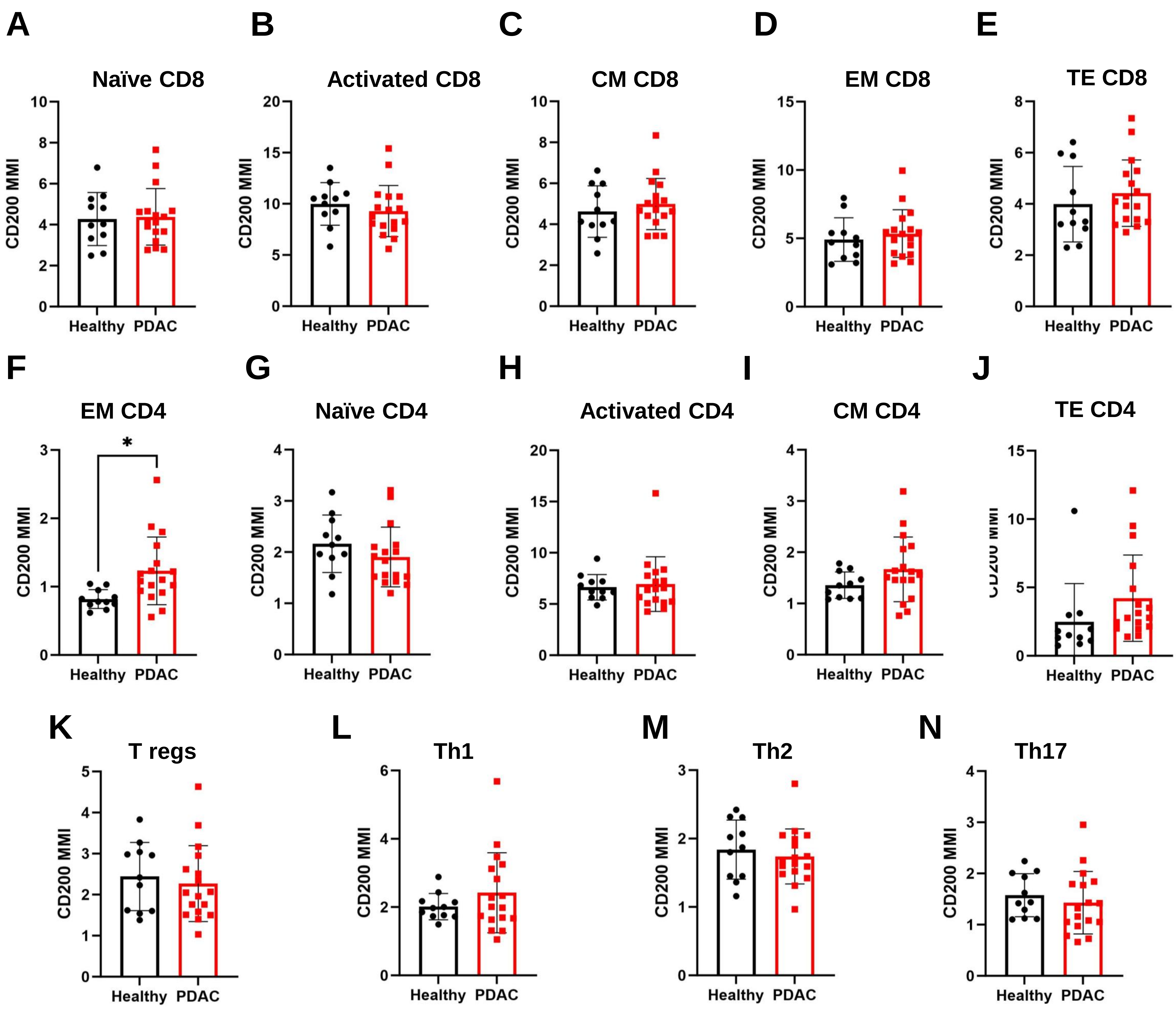

Supplemental Figure 3: CD200 expression on specific T cell populations. PBMCs were isolated from PDAC patients (n = 17) and healthy donors (n = 12) and stained with a panel of 38 antibodies for CyTOF. MMI of CD200 was quantified on **A** naïve, **B** activated, **C** CM, **D** EM, and **E** TE CD8 T cells. MMI of CD200 on **F** EM, **G** naïve, **H** activated, **I** CM, **J** TE CD4 T cells. MMI of CD200 on **K** T regs, **L** Th1, **M** Th2, and **N** Th17. Means ± STD, * = $p < 0.05$

Supplemental Table 1. Metal-isotope antibodies

Marker	Metal
CD45	89Y
CD196/CCR6	141Pr
CD123	143Nd
CD19	144Nd
CD4	145Nd
CD8a	146Nd
CD11c	147Sm
CD16	148Nd
CD45RO	149Sm
CD45RA	150Nd
CD161	151Eu
CD194/CCR4	152Sm
CD25	153Eu
CD27	154Sm
CD57	155Gd
CD183/CXCR3	156Gd
CD185/CXCR5	158Gd
CD28	160Gd
CD38	161Dy
CD56/NCAM	163Dy
TCRγδ	164Dy
CD294	166Er
CD197/CCR7	167Er
CD14	168Er
CD3	170Er
CD20	171Yb
CD66b	172Yb
HLA-DR	173Yb
IgD	174Yb
CD127	176Yb
CD11b/Mac-1	209Bi
CD33	165Ho
Viability	103Rh

Supplemental Table 2. Characteristics of Patients

	Tissue Diagnosis	*Normal*	*Cancer Adjacent*	*Pancreatic Cancer*
**Total**		18	27	127
**Sex**	*Female*	8	15	53
	Male	10	12	74
**Age**	*< 53 yrs*	18	11	62
	> 53 yrs	0	16	65
**Grade**	*1*	-	-	32
	2	-	-	31
	3	-	-	37
**Stage**	*I*	-	-	48
	II	-	-	71
	III/IV	-	-	6

Supplemental Table 3. Genetic identification of iCAFs vs myCAFs

iCAFs	myCAFs
C3	MYL9
DUSP1	CALD1
FBLN1	MMP11
LMNA	HOPX
CLU	BGN
CCDC80	IGFBP7
MYC	TPM2
EFEMP1	CTHRC1
HAS1	ACTA2
NR4A1	TAGLN
CFD	INHBA
ANXA1	COL10A1
CXCL12	TPM1
FGF7	POSTN
KLF4	GRP
EMP1	CST1
GPRC5A	
SRPX	
MT2A	
MEDAG	
IGF1	
MGST1	
MCL1	
CEBPD	
S100A10	
UAP1	
TNXB	
CEBPB	
PNRC1	
SOCS3	
PTGDS	
FOSB	
NFKBIA	
CXCL2	
THBS1	
CCL2	
OGN	
GSN	
DPT	
PLA2G2A	
NAMPT	
ITM2A	
RGCC	
JUND	
NNMT	
ZFP36	
PIM1	
CPE	
GFPT2	
SOD2	
KDM6B	
FSTL1	
FBLN2	
NR4A3	
MFAP5	
ABL2	
SGK1	
CILP	
UGDH	
FBLN5	
ADAMTS1	
ADH1B	
WISP2	
GPX3	
S100A4	
IL6	
HAS2	
PLAC9	
IGFBP6	
FBN1	
BDKRB1	
TPPP3	
RASD1	
MT1A	
CXCL14	
PI16	
APOE	
IL8	
ARC	
PTX3	
TNFAIP6	
MT1E	
MT1X	
CXCL1	
